# The Toxic Effects of Tetrachlorobisphenol A in *Saccharomyces cerevisiae* Cells via Metabolic Interference

**DOI:** 10.1038/s41598-017-02939-y

**Published:** 2017-06-01

**Authors:** Juan Tian, Zhihua Ji, Fengbang Wang, Maoyong Song, Hao Li

**Affiliations:** 1 Beijing Key Laboratory of Bioprocess, College of Life Science and Technology, Beijing University of Chemical Technology, Beijing, 100029 China; 2 State Key Laboratory of Environmental Chemistry and Ecotoxicology, Research Center for Eco-Environmental Sciences, Chinese Academy of Sciences, Beijing, 100085 China; 3 Institute of Environment and Health, Jianghan University, Wuhan, 430056 China

## Abstract

Tetrachlorobisphenol A (TCBPA) is a common flame retardant detected in different environments. However, its toxic effects on animals and humans are not fully understood. Here, the differential intracellular metabolites and associated gene expression were used to clarify the metabolic interference of TCBPA in *Saccharomyces cerevisiae*, a simple eukaryotic model organism. The results indicated that TCBPA treatment promoted the glycolysis pathway but inhibited the tricarboxylic acid (TCA) cycle, energy metabolism and the hexose monophosphate pathway (HMP) pathway. Thus, the HMP pathway produced less reducing power, leading to the accumulation of reactive oxygen species (ROS) and aggravation of oxidative damage. Accordingly, the carbon flux was channelled into the accumulation of fatty acids, amino acids and glycerol instead of biomass production and energy metabolism. The accumulation of these metabolites might serve a protective function against TCBPA stress by maintaining the cell membrane integrity or providing a stable intracellular environment in *S*. *cerevisiae*. These results enhance our knowledge of the toxic effects of TCBPA on *S*. *cerevisiae* via metabolic interference and pave the way for clarification of the mechanisms underlying TCBPA toxicity in animals and humans.

## Introduction

With increasing public awareness of fire prevention and fire safety standards for products, fire-retardant materials have been widely used on a global scale. Halogenated flame retardants have several advantages, including good performance, stability and a robust production method, making them the world’s most popular type of organic flame retardant^1–3^.

Tetrachlorobisphenol A (TCBPA), which contains chlorine atoms on the phenolic rings of bisphenol A (BPA), is a widely used halogenated flame retardant^4, 5^. TCBPA is also used as an additive in plastics, building materials, and synthetic textiles^6, 7^. However, halogenated organic compounds, including TCBPA, that are released from flame-retardant materials could contaminate the soil, water, sediment and other environmental media, thereby entering the human body, animals, plants and other biological media through various routes^6, 8, 9^. The long-term accumulation of TCBPA can reach toxic concentrations through the bio-magnification of the food chain and could further cause some adverse effects on wildlife and human beings because of its persistence, lipophilic characteristics and bioaccumulation^2, 10, 11^. It has been found that TCBPA and tetrabromobisphenol A (TBBPA) can function as candidate endocrine disruptors, thyroid hormone-disrupting chemicals and peroxisome proliferator activated receptor gamma (PPARγ) agonists^6, 12–14^. Moreover, the ability of TCBPA to activate the human pregnane X receptor (hPXR) is superior to those of TBBPA and BPA^7^. Song *et al*.^15^ indicated that the toxicity order of halogenated BPAs on zebrafish was TCBPA > TBBPA > BPAF^15^. However, besides the abovementioned adverse effects, it is not known if there are other toxic effects of TCBPA.

*Saccharomyces cerevisiae*, which is a commonly used eukaryotic model microorganism, has some advantages compared with animals and humans, such as its simple structure, fully interpreted genetic background and ease of manipulation. Indeed, as a simple single cell, *S*. *cerevisiae* has been used as a model for detecting drug exposure-associated toxic effects to quickly provide functional clues and pave the way for more complex studies in animals or humans. For example, *S*. *cerevisiae* has been used as a useful eukaryotic model to study human diseases related to transition metal ions (i.e., iron and copper)^16^, human neurodegenerative disorders^17^, as well as to discover anticancer drugs^18^. Moreover, a series of recombinant yeast strains has been used to clarify the effect of phenolic compounds on endocrine systems^19, 20^.

In our previous studies using a gas chromatography-mass spectrometry (GC-MS)-based metabolomics strategy, it was found that external stress could cause intracellular metabolite changes in *S*. *cerevisiae* ^21, 22^. Metabolomics results can reflect a series of metabolite changes in a biological system secondary to external stimuli or pathological insult^23, 24^, and the metabolite content or composition changes can directly reveal the phenotype changes that occurred in the living system. Therefore, *S*. *cerevisiae* might be an ideal model to investigate TCBPA toxic effects associated with metabolic interference.

Considering that TCBPA might cause several metabolic perturbations and might be associated with many genes, mRNAs, proteins and metabolites, a GC-MS-based metabolomics strategy combined with multivariate statistical and gene expression analyses was used to determine the TCBPA exposure-associated intracellular metabolic changes in *S*. *cerevisiae*. These results helped elucidate the mechanisms involved in TCBPA toxicity to yeast and may indicate further effects in other animals and humans.

## Results

### TCBPA exposure-associated *S*. *cerevisiae* cytotoxicity

**Figure 1:**
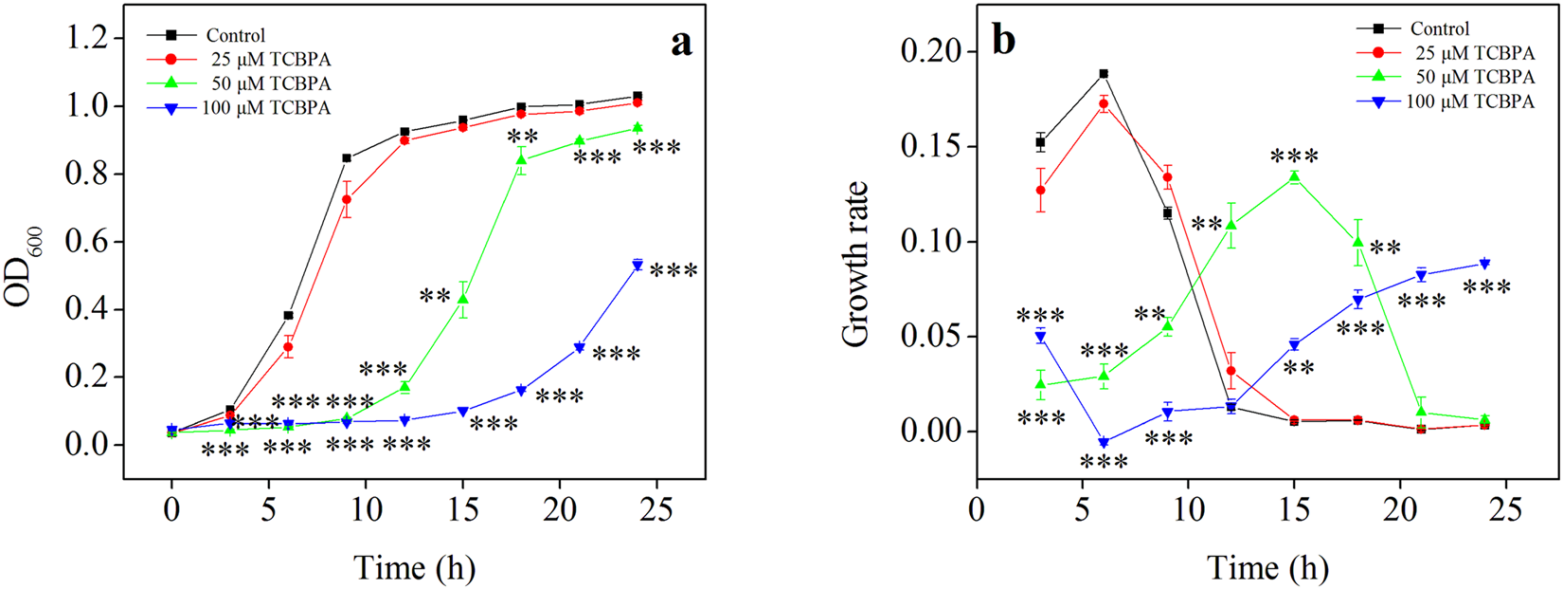
Effect of different concentrations of TCBPA on the growth of *S*. *cerevisiae*. (**a**) Growth curve. (**b**) Growth rate. *P < 0.05 compared with the control group; **P < 0.01 compared with the control group; ***P < 0.001 compared with the control group.

When *S*. *cerevisiae* was cultured in the presence of a low concentration of TCBPA, the yeast cell growth was not noticeably inhibited (*P* > 0.05) (Fig. 1a). However, the addition of an intermediate or high concentration of TCBPA inhibited the growth of yeast cells (*P* < 0.05), and the inhibition degree increased as the TCBPA concentration increased (Fig. 1a). The growth rate of *S*. *cerevisiae* treated with a low concentration of TCBPA also showed no difference from that of the control group (without TCBPA) (*P* > 0.05), and both reached a maximum after 6 h of cultivation (Fig. 1b). However, the delayed growth rate peak after 15 h of cultivation under intermediate and high concentrations of TCBPA exposure indicated a TCBPA treatment-associated cell cycle delay.

### Effect of TCBPA on the intracellular metabolite profiles of *S*. *cerevisiae*

For metabolomics analysis, *S*. *cerevisiae* cells were harvested after 8 h of incubation. Typical GC-MS TIC chromatograms from the *S*. *cerevisiae* cells treated with various concentrations of TCBPA are shown in Supplementary Fig. S1. The PCA PC1/PC2 scores plot was constructed to represent the sample distribution in the new multivariate space (*R* ^2^ *X*_cum_ = 0.917, *Q* ^2^_cum_ = 0.807). A clear discrimination was observed among the control and TCBPA-treated groups (Supplementary Fig. S2a). This plot suggests that 91.7% of the variation in the data set was due to the TCBPA treatment, demonstrating that TCBPA is likely responsible for the metabolic perturbation observed in the data set. To confirm the TCBPA-associated variations in the metabolic variations, the class separation was further optimized using the PLS-DA model (*R* ^2^ *X*_cum_ = 0.764, *Q* ^2^_cum_ = 0.615), and good discriminability was also observed (Supplementary Fig. S2b).

### Changes in the intracellular metabolites at each TCBPA concentration

**Figure 2:**
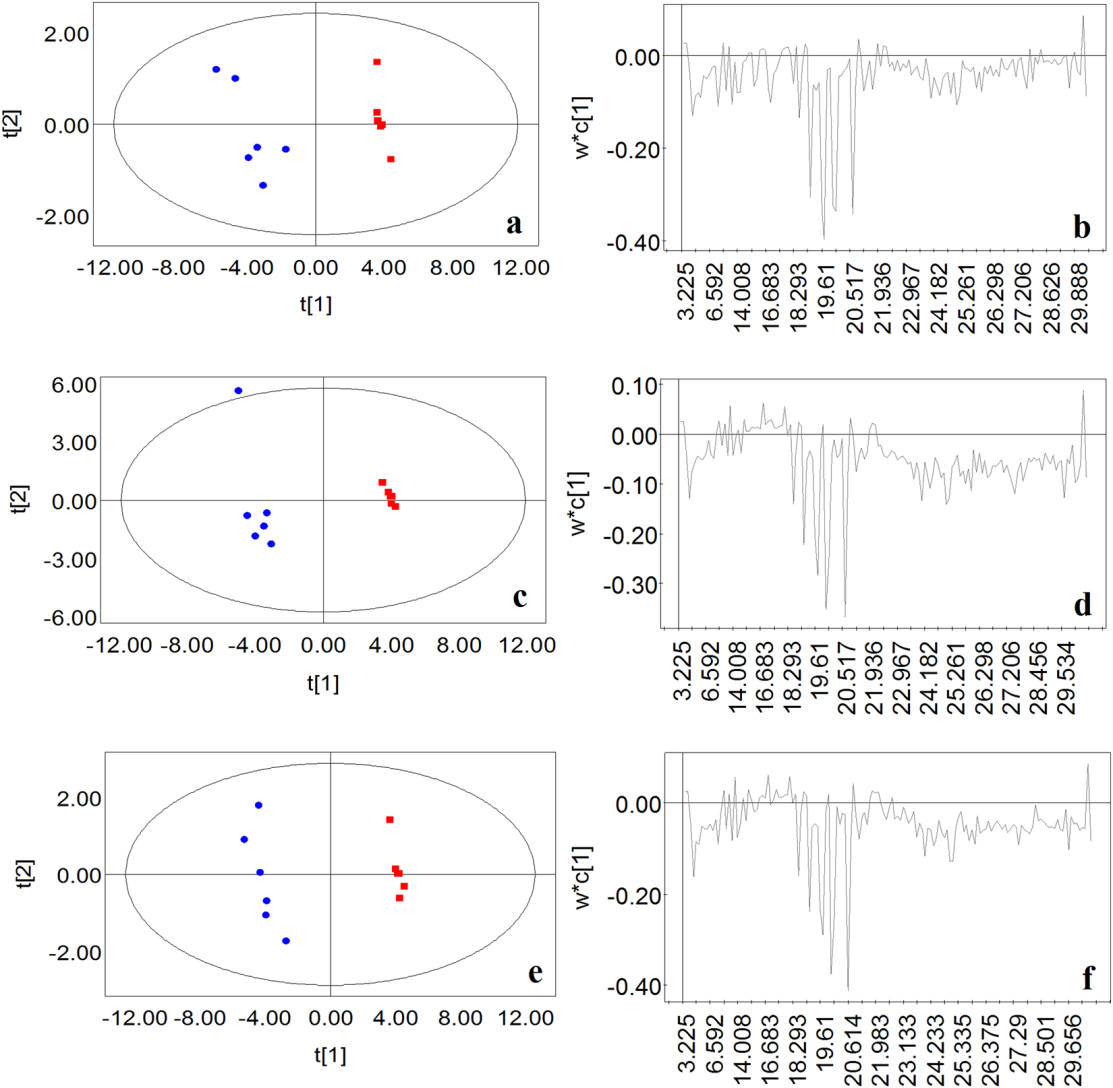
PLS-DA model plots for the control group (red symbols) versus the groups treated with different concentrations of TCBPA (blue symbols). Cross-validated score plots of the pairwise comparison between the (**a**) control versus 25 μM, (**c**) control versus 50 μM, and (**e**) control versus 100 μM TCBPA-treated groups. Two groups in each score plot were separated along the first component. In the score plots, the confidence interval is defined by the Hotelling’s T2 ellipse (95% confidence interval), and observations outside the confidence ellipse are considered outliers. Loading plots of the pairwise comparison between the (**b**) control versus 25 μM, (**d**) control versus 50 μM, (**f**) control versus 100 μM TCBPA-treated groups. The metabolites with the largest intensities contributed to the clustering.

The PLS-DA pairwise comparison between the control group and each TCBPA-treated group suggested an obvious metabolic difference between the classes in each pairwise comparison with respect to the first component (Fig. 2a,c and e). The PLS-DA models were well constructed with an excellent fit and satisfactory predictive power (Supplementary Table S1). The major metabolic perturbations that cause these discriminations were identified from the line plots of the *X*-loadings of the first component of the PLS-DA models (Fig. 2b,d and f).

A metabolite with a VIP value greater than 1 demonstrates a significant contribution to the separation of groups within the PLS-DA models^25^. The VIP plots demonstrated the order of identified metabolites contributed to the class separation (Supplementary Fig. S3). Based on the *X*-loadings line plots and VIP plots, 31 paired retention time-mass to charge ratio (RT-M/Z) variables contributing to the separation of the control group and TCBPA-treated groups were selected according to the cutoff VIP value (VIP > 1) (Table 1).

**Table 1:** The intracellular metabolites of *Saccharomyces cerevisiae* identified by GC-MS that differ between the control group and the TCBPA-treated groups.

retention time	Metabolite	TCBPA content (μM)			
		0	25	50	100
6.592	D-Lactic acid	31.12 ± 4.22	37.59 ± 2.39	34.29 ± 2.03	56.14 ± 4.99**
7.971	L-Alanine	22.22 ± 7.17	80.65 ± 7.25***	46.42 ± 12.76	74.03 ± 6.69***
11.061	Phosphoric acid	4.20 ± 0.82	0*	0**	0**
12.315	L-Valine	9.85 ± 2.62	37.01 ± 2.67***	17.21 ± 5.94	30.49 ± 2.74***
13.263	Serine	2.56 ± 0.70	0*	0*	0*
13.611	Leucine	12.61 ± 2.75	61.97 ± 4.25***	31.27 ± 9.82	52.83 ± 4.37***
13.756	Glycerol	38.32 ± 3.10	69.85 ± 4.48***	52.27 ± 5.00*	72.04 ± 2.78***
14.008	L-Isoleucine	11.13 ± 2.53	40.84 ± 3.26***	12.75 ± 8.03	28.47 ± 4.60**
14.199	Glycine	3.59 ± 1.18	5.25 ± 1.77	8.73 ± 1.75*	10.12 ± 2.03*
14.3	Succinic acid	4.72 ± 0.76	3.57 ± 1.67	0**	0**
15.457	L-threonine	9.34 ± 2.54	22.79 ± 2.05**	4.89 ± 4.89	13.54 ± 3.70
16.889	L-Proline	25.02 ± 5.65	76.25 ± 6.40***	15.67 ± 15.67	27.85 ± 11.85
18.293	Glutamic acid	2.61 ± 0.87	0*	0*	0*
18.987	Phosphoric acid	4.18 ± 1.47	0*	0*	0*
19.252	Glucofuranoside	5.09 ± 3.63	438.34 ± 49.83***	319.60 ± 86.23*	342.80 ± 56.12**
19.47	D-Fructopyranose	2.13 ± 0.74	16.83 ± 1.18***	15.10 ± 1.45***	17.77 ± 0.58***
19.578	D-Talopyranose	2.07 ± 2.07	512.15 ± 50.97***	276.10 ± 81.90*	323.98 ± 57.16**
19.61	D-Allofuranose	12.21 ± 3.24	728.37 ± 70.44***	517.09 ± 135.92*	521.96 ± 91.76**
19.836	Tyrosine	2.68 ± 1.03	7.71 ± 1.69*	0*	0*
19.973	D-Galactopyranose	53.63 ± 4.99	541.25 ± 51.57***	652.36 ± 30.51***	818.21 ± 61.67***
20.039	D-Talopyranose	5.98 ± 1.88	508.52 ± 35.71***	378.44 ± 80.32**	405.68 ± 54.30**
20.517	D-Allopyranose	90.93 ± 8.08	644.93 ± 74.87**	740.30 ± 28.26***	1014.02 ± 81.57***
21.388	Mannobiose	2.60 ± 1.73	37.53 ± 11.81*	17.97 ± 3.69**	16.90 ± 1.50***
23.282	Acetic acid	3.19 ± 1.80	9.97 ± 2.28*	20.01 ± 4.29**	15.82 ± 1.51***
23.642	Cholesterol	12.77 ± 1.68	33.98 ± 9.73	58.74 ± 9.34**	51.53 ± 9.36**
23.902	Hexadecanoic acid	19.66 ± 3.41	72.24 ± 16.17*	111.20 ± 11.04***	105.21 ± 17.22**
24.109	Sucrose	3.28 ± 1.16	15.93 ± 3.50**	23.19 ± 5.63**	22.14 ± 2.61***
24.233	Palatinose	4.03 ± 1.08	37.59 ± 2.94***	62.31 ± 5.32***	54.43 ± 5.74***
24.876	Octadecanoic acid	22.33 ± 4.90	80.40 ± 10.80**	126.02 ± 11.82***	116.79 ± 11.05***
24.989	Trehalose	6.16 ± 1.43	37.94 ± 1.80***	90.76 ± 11.13**	109.02 ± 20.31**
27.423	Unknown	6.68 ± 1.27	31.02 ± 3.69**	87.42 ± 14.58**	61.94 ± 11.79**

The VIP scores of all listed metabolites are greater than 1. The data represents the relative peak intensity and are presented as the mean ± SEM. *P < 0.05 compared with the control. **P < 0.01 compared with the control. ***P < 0.001 compared with the control.

As shown in Supplementary Fig. S4, the HCA plot of the 31 identified differential metabolites reflected a clustering pattern that was similar to the PLS-DA analysis results. The intermediate and high concentrations of the TCBPA-treated groups were separated from the control and low concentration TCBPA-treated groups. The HCA results were consistent with the PLS-DA models and also verified the predictive accuracy of the PLS-DA models.

### Effect of TCBPA on gene expression

**Figure 3:**
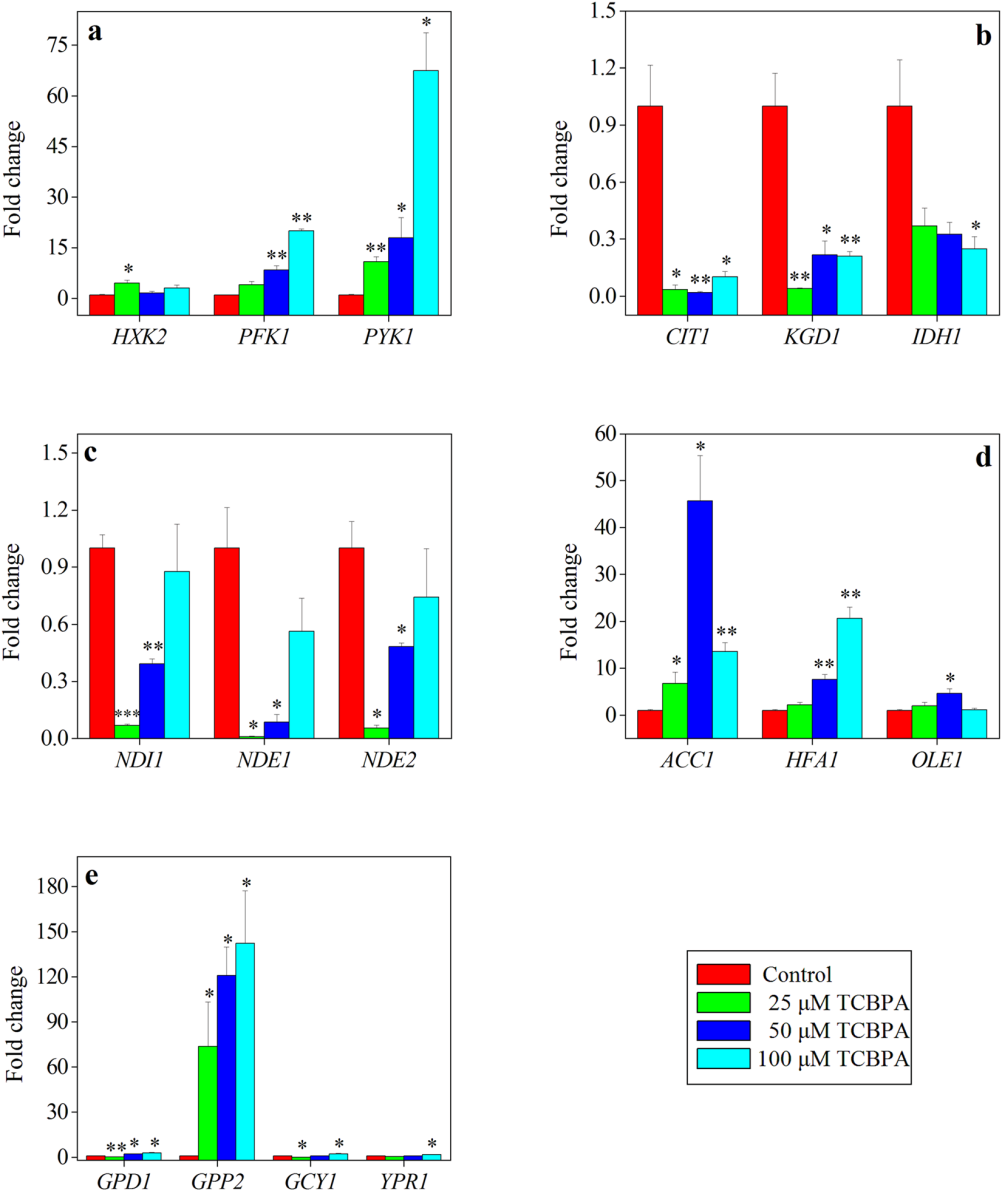
Change in the transcriptional levels of genes participating in the main metabolic pathway of *S*. *cerevisiae* between the control group and TCBPA-treated groups. (**a**) EMP pathway; (**b**) TCA cycle; (**c**) redox pathway; (**d**) fatty acid pathway; (**e**) glycerol pathway. All results were normalized to the *ACT1* transcriptional level in the same sample. *P < 0.05 compared with the control group. **P < 0.01 compared with the control group. ***P < 0.001 compared with the control group.

Compared with that in the control group, the expression of *HXK2*, which encodes the rate-limiting enzyme involved in the Embden-Meyerhof-Parnas (EMP) metabolic pathway, was increased in the low TCBPA concentration treated group (*P* < 0.05), while it did not change in the intermediate and high TCBPA concentration treated groups (*P* > 0.05) (Fig. 3a). The expression of two other key EMP pathway genes, *PFK1* and *PYK1*, was continuously increased as the TCBPA concentration was increased (*P* < 0.05) (Fig. 3a).

Compared with that in the control group, the expression of *CIT1*, *KGD1*, and *IDH1* (Fig. 3b), which encode the rate-limiting enzymes involved in the tricarboxylic acid (TCA) cycle, decreased in the TCBPA-treated groups (*P* < 0.05) (Fig. 3b). Additionally, compared with the control group, the low and intermediate TCBPA concentration treated groups had lower transcriptional levels of *NDI1*, *NDE1* and *NDE2*, which are responsible for encoding NADH dehydrogenase in the mitochondrial, inner and outer membranes, respectively (*P* < 0.05) (Fig. 3c). However, no expression changes in NADH dehydrogenase genes were detected between the control group and high concentration TCBPA treated group (*P* > 0.05) (Fig. 3c). Treatment of TCBPA at a high concentration significantly inhibited yeast cell growth (Fig. 1a) and, to some extent, would likely cause cell distortion or even disruption, thereby leading to RNA degradation. Although the RNA integrity might be affected, the expression of genes in the high concentration TCBPA treated group was still used to examine trends.

The expression of *ACC1* and *HFA1*, which encode rate-limiting enzymes in the synthesis of fatty acids and unsaturated fatty acids (UFAs), increased in the TCBPA-treated groups (*P* < 0.05) (Fig. 3d). However, the expression of another key gene, *OLE1*, was only increased in the group treated with the intermediate concentration of TCBPA (*P* < 0.05) (Fig. 3d).

Moreover, compared with the control group, the TCBPA-treated groups had higher transcriptional levels of *GPD1* and *GPP2*, which encode rate-limiting enzymes involved in glycerol metabolism (*P* < 0.05) (Fig. 3e). However, the transcriptional levels of two other genes, *GCY1* and *YPR1*, showed almost no changes except for increases in the group treated with the high concentration of TCBPA (Fig. 3e).

## Discussion

Extensive industrial production and household application facilitate the release of TCBPA into the environment. Several studies have shown that it has been detected in soils, rivers and even the blood of animals and humans^8, 26–28^. Although numerous studies have implied that TCBPA has endocrine-disrupting, thyroid hormone-disrupting and peroxisome proliferator-activated effects^6, 13^, its exact toxicity is not clearly defined. Additionally, TCBPA was reported to inhibit the proliferation and survival of cells^29, 30^. Here, our results showed that TCBPA inhibited yeast cell growth in a dose-dependent manner (Fig. 1a). Moreover, the changes in the growth rate suggested that TCBPA exposure prolonged the cell cycle (Fig. 1b). Several studies have revealed that the metabolic changes involved a decreased biomass and a prolonged cell cycle^31, 32^. In addition, research in Daphnia and in rats suggested another flame retardant, Firemaster 550, might also cause metabolic interference^33, 34^. In this study, our metabolomics results revealed TCBPA treatment-associated metabolic perturbations, implying a potential toxic effect of TCBPA on organisms. Therefore, the metabolic interference and cytotoxicity of TCBPA were systematically studied using a GC-MS-based metabolomics strategy combined with multivariate and gene expression analyses using *S*. *cerevisiae* as a model.

**Figure 4:**
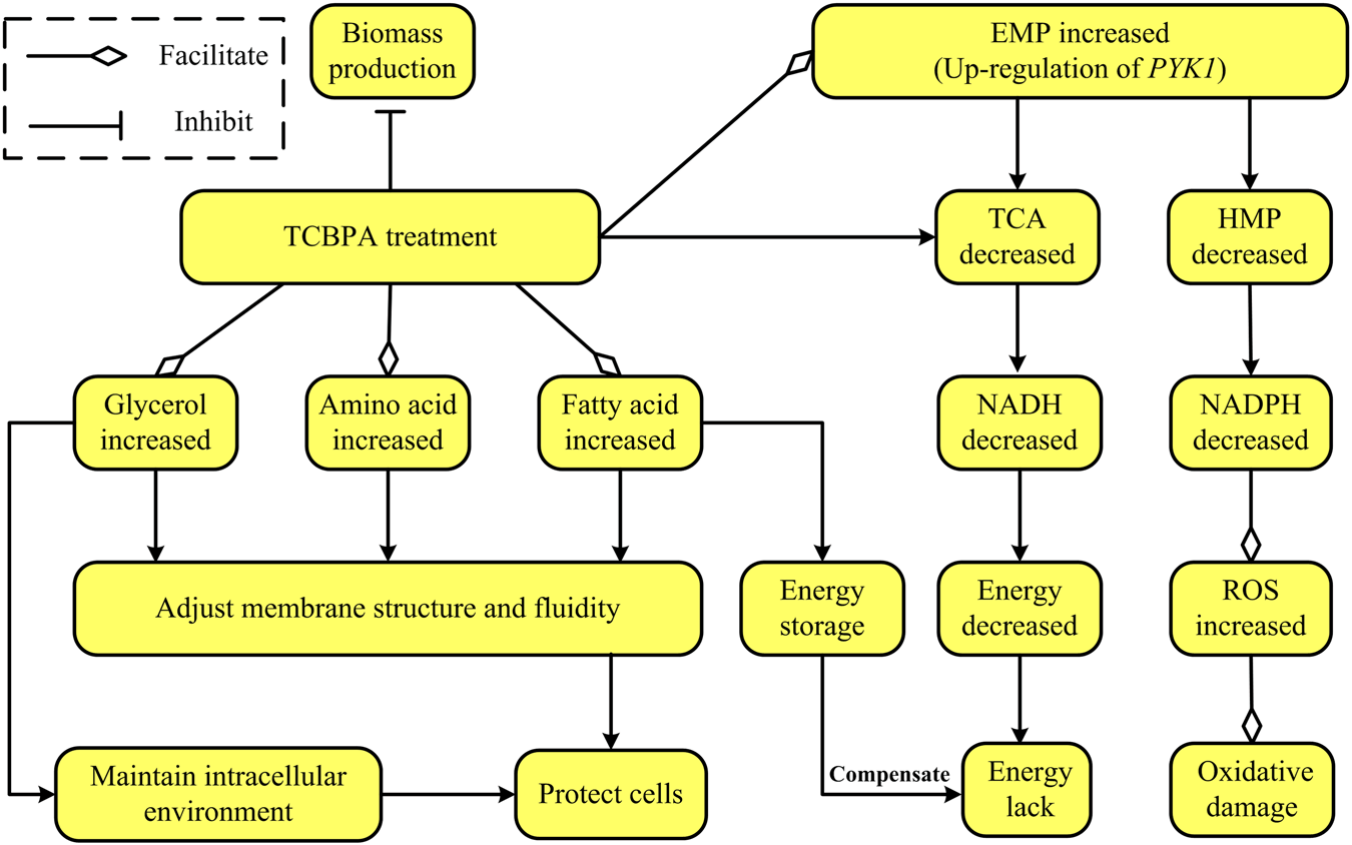
Overview of the effect of TCBPA treatment on metabolic changes in *Saccharomyces cerevisiae*.

Compared with the control group, the amounts of galactopyranose, mannobiose and fructopyranose were increased in all TCBPA-treated groups (*P* < 0.05) (Table 1, Supplementary Fig. S5), demonstrating that glycolysis might be promoted by TCBPA treatment, increasing the usage of more carbon sources. The higher expression of three key genes (i.e., *HXK2*, *PFK1* and *PYK1*) involved in the EMP pathway in *S*. *cerevisiae* cells also confirmed the activation of the EMP pathway in the TCBPA-treated groups. Interestingly, at first glance, an activated EMP pathway contradicted the TCBPA treatment-associated biomass reduction. These preliminary results indicated that glycolytic flux might be rerouted from the biomass to the production of other compounds in accordance with the inhibition of *S*. *cerevisiae* cell growth (Fig. 4). However, it was not clear which metabolic pathway(s) the carbon flux was redistributed into.

Activation of the EMP pathway also suggested that further activation of the TCA cycle might be realized with TCBPA treatment. However, the TCBPA treatment-associated decrease in the content of succinic acid (Table 1, Supplementary Fig. S5) and lower expression of three key TCA cycle genes (i.e., *CIT1*, *KGD1* and *IDH1*) (Fig. 3) indicated the inhibition of the TCA cycle by TCBPA treatment in *S*. *cerevisiae* cells. This would be in agreement with the higher expression of *PYK1* in the EMP pathway promoted by TCBPA treatment (Fig. 3a). The up-regulation of *PYK1* in the EMP pathway might lead to less carbon flux to the TCA cycle, and less energy would be generated^35^. The TCA cycle is an important way for energy to be supplied for the growth processes of *S*. *cerevisiae*. A large amount of NADH-reducing power could be produced through the TCA cycle and could be subsequently transferred into the electron transport chain to produce ATP. Inhibition of the TCA cycle because of TCBPA treatment would undoubtedly decrease the amount of NADH produced. Moreover, NADH and NAD^+^ cannot be directly transported across the mitochondrial membrane in *S*. *cerevisiae* cells, and the NADH in the mitochondria and cytoplasm needs to be reoxidized by NADH dehydrogenase encoded by *NDI1*, *NDE1* and *NDE2*, respectively. In this study, the down-regulation of *NDI1*, *NDE1* and *NDE2* (Fig. 3) indicated the inactivation of the reoxidation of NADH to NAD^+^, a finding that was also consistent with the decreased NADH content. The decreased reoxidation of NADH to NAD^+^, together with the inhibition of the TCA cycle in the TCBPA-treated groups, indicated that energy metabolism was inhibited by TCBPA treatment (Fig. 4).

The up-regulation of *PYK1* might also control the metabolic flux switching from fermentation to respiration^36^, as well as the redistribution of carbon flux from the hexose monophosphate pathway (HMP) to glycolysis. The reducing power, NADPH, which can play vital roles in antioxidant defence by acting as an essential cofactor for thioredoxin- or glutathione-dependent enzymes that constitute the main cellular defences against oxidative stress^37^, is mainly produced by a glucose dehydrogenation reaction through the HMP pathway^38^. In the presence of TCBPA, the HMP flux was decreased; therefore, less NADPH was produced than in the control group. A decrease in the amount of generated NADPH would provide less reducing equivalents for intracellular biosynthetic processes and antioxidant defence action^39^, thereby leading to the accumulation of reactive oxygen species (ROS) (Fig. 4). In fact, TCBPA treatment did induce the accumulation of ROS in yeast cells (Supplementary Fig. S6). Accumulated ROS could induce apoptosis^40^ and are normally accompanied by a decreased mitochondrial membrane potential, which is essential for ATP production^41^, further contributing to decreased energy production.

Given the activated EMP pathway and inactivated TCA cycle, the carbon source may flow to other metabolic pathways such as those responsible for the metabolism of fatty acids and amino acids (Fig. 4). In fact, TCBPA treatment did increase the levels of fatty acids (i.e., hexadecanoic acid (C_16:0_) and octadecanoic acid (C_18:0_)) (Table 1, Supplementary Fig. S5), which were indirectly converted from pyruvate. The up-regulation of fatty acid synthetic genes (i.e., *ACC1* and *HFA1*) (Fig. 3) also supported the increase in the amounts of hexadecanoic and octadecanoic acids in the TCBPA-treated groups. Fatty acid is an important component of the cell membrane and plays a self-protective role in response to external stimuli; increased levels of fatty acids, especially saturated fatty acids (SFAs), can maintain the cell membrane integrity through decreasing the membrane fluidity to avoid drastic TCBPA stress-associated changes in the membrane structure, thereby enhancing the tolerance of *S*. *cerevisiae* cells to stress^21^. The increased fatty acid was also consistent with the previously reported lipid accumulation observed for TBBPA- or TCBPA-treated zebrafish and murine 3T3-L1 cells^13, 14^. Moreover, decreased energy metabolism and increased fatty acid content were closely related to cytotoxicity and the occurrence of cancer. For example, radioresistant rSCC-61 cells were used to study head and neck squamous cell cancer (HNSCC), and Human leukemia cells K562 and KCL-22 were used to study the treatment of Human leukemia by anti-cancer fatty-acid derivative AIC-47, indicating that fatty acids/fatty-acid derivative and energy metabolism are associated with cancer^42, 43^. The accumulated fatty acids could also function in energy homeostasis in that excess carbon sources may be converted to fatty acids for energy storage in cancer cells^44, 45^. The accumulated saturated fatty acids in this study may also function as an energy storage to compensate for the lack of energy in the TCA cycle to some extent (Fig. 4).

Additionally, the levels of valine, leucine, isoleucine, alanine, glycine, lactate and glycerol, which can be converted from two EMP intermediates, pyruvate and dihydroxyacetone phosphate, were increased in the TCBPA-treated groups (Table 1, Supplementary Fig. S5). Leucine, isoleucine and valine are all branched-chain amino acids (BCAAs). It is known that *Bacillus subtilis* cell membrane fluidity changes occur through the biosynthesis of BCAAs^46^. Yeast cells might also adjust their membrane fluidity by increasing BCAAs in response to TCBPA stress. Moreover, the presence of these amino acids may enhance the ethanol tolerance of *S*. *cerevisiae* cells by stabilizing their membrane structure^47^. Increased levels of these amino acids, together with the other increased amino acids, might also confer tolerance on *S*. *cerevisiae* to TCBPA stress (Fig. 4).

Glycerol, an important cytoprotectant and osmotolerant compound, may help to balance the intracellular and extracellular environment under conditions of hyperosmosis in *S*. *cerevisiae* ^48, 49^. Glycerol may act as an opportune compound to promote the robustness of the yeast cell when encountering ethanol stress^50^. Similarly, up-regulation of the genes *GPD1* and *GPP2* (Fig. 3) promoted the accumulation of glycerol, which, in turn, may play a protective role to maintain a stable cellular environment for *S*. *cerevisiae* and counteract TCBPA stress. Moreover, glycerol could accumulate at the expense of the biomass enhancement^51^. In this study, the increased glycerol content secondary to TCBPA treatment might also demonstrate that glycolytic flux was rerouted from biomass production to the accumulation of glycerol in accordance with the inhibition of *S*. *cerevisiae* cell growth (Fig. 4).

In summary, this study clarified the usefulness of a GC-MS-based metabolomics strategy to evaluate the toxic effects of TCBPA on *S*. *cerevisiae* cells. The changes in the intracellular metabolites and expression of genes involved in the central metabolic pathways in the TCBPA-treated groups compared with the control group suggested that the core metabolic fluxes of *S*. *cerevisiae* were affected by TCBPA treatment. In the presence of TCBPA, the glycolysis pathway was activated, while the TCA cycle, energy metabolism and HMP pathway were inhibited. A decrease in the HMP pathway produced less reducing power, thereby contributing to the accumulation of ROS and subsequently exacerbating oxidative damage. Accordingly, the carbon flux was channelled into the synthesis of fatty acids, amino acids and glycerol, and the accumulation of these metabolites might function as protective agents against TCBPA stress by maintaining membrane integrity and a stable intracellular environment. These results enhance our knowledge of the toxic effects of TCBPA on *S*. *cerevisiae* cells and should provide a foundation for clarifying the mechanisms involved in TCBPA toxicity in animals and humans.

## Materials and Methods

### Strains, media and culture conditions

The *S*. *cerevisiae* S288c strain (ATCC number 204508) used in this study was purchased from the China General Microbiological Culture Collection Center (Beijing, China). *S*. *cerevisiae* S288c was pre-cultured in YPD broth (2% glucose, 2% peptone, 1% yeast extract) at 30 °C for 18 h. The pre-cultured *S*. *cerevisiae* cells were inoculated into fermentative medium, and the initial optical absorbance of 600 nm was adjusted to 0.1. The fermentative culture was performed at 30 °C with shaking at 150 rpm in 250 ml cotton-plugged flasks containing 100 ml of fresh YPD broth with or without TCBPA. TCBPA was dissolved in DMSO. For the TCBPA-treated cells, 50, 100, or 200 μl of 50 mmol/L TCBPA (i.e., corresponding to the TCBPA final concentration of 25 μM, 50 μM or 100 μM, respectively) was added to the YPD broth at the beginning of the primary culture. In the subsequent experiments, 25, 50, and 100 μM TCBPA-treated cells were named as the low, intermediate and high concentration TCBPA-treated groups, respectively.

### Determination of the growth curve

Samples (700 μl) were taken after 0, 3, 6, 9, 12, 15, 18, 21 and 24 h of incubation. The *S*. *cerevisiae* cell growth curve was determined by measuring the optical density at 600 nm with a 752 visible spectrophotometer (Shanghai Optical Instrument Factory, Shanghai, China). The experiment was conducted in triplicate. The growth rate (μ) was calculated using OD_600_ values according to the following equation:

$${\rm{\mu }}=(\mathrm{ln}\,{{X}}_{2}-\,\mathrm{ln}\,{{X}}_{1})/({t}_{2}-{t}_{1})$$

*X*_1_ and *X*_2_ refer to the OD_600_ values of the cell cultures at the culture times *t*_1_ and time *t*_2_, respectively.

### Preparation of metabolome samples

Metabolome samples of *S*. *cerevisiae* were prepared according to our previously published procedure^21^. Briefly, six biological replicates were performed for each group. Two millilitres of each culture was quickly harvested after 8 h of incubation and were immediately transferred to 15 ml tubes containing 8 ml of −40 °C pre-chilled 60% methanol to quench the culture. After quenching, the yeast cells were collected by centrifugation (10,000 × *g*, −4 °C, 10 min). The supernatant was discarded, and the pellet spiked with an internal standard (50 μl of ribitol in water, 0.5 mg/ml) was prepared for the extraction of intracellular metabolites. The samples were suspended in 0.75 ml of −40 °C pre-chilled pure methanol and then were frozen in liquid nitrogen. The frozen suspension was thawed in an ice bath, and this freeze-thaw process was repeated three times before centrifugation (10,000 × *g*, −4 °C, 10 min). The supernatant was collected, and an additional 0.75 ml of pre-chilled pure methanol was added to the pellet. The mixture was vortexed for 30 s prior to centrifugation (10,000 × *g*, −4 °C, 10 min). Both supernatants were pooled together and were stored at −20 °C until use.

### Sample derivatization

The samples were dried in a vacuum centrifuge dryer at 30 °C for 24 h. For derivatization, 100 μl of methoxyamine hydrochloride in pyridine (20 mg/ml), the first derivatizing agent, was added to the dried samples prior to incubation at 30 °C for 2 h. One hundred microlitres of N-methyl-N-(trimethylsilyl) trifluoroacetamide (MSTFA), the second derivatizing agent, was added to the samples before incubation at 30 °C for 3 h to trimethylsilylate the polar functional groups. The derivatized samples were rapidly filtered through 0.22-μm-diameter round hydrophobic nylon filters, and the samples were diluted by one-third with acetonitrile before GC-MS analysis.

### Metabolomics analysis

The changes in the metabolites in *S*. *cerevisiae* with and without TCBPA treatment were analysed using GC-MS. Chromatography analysis was performed on a 7890B-5977A GC/MSD solution system (Agilent Co., Santa Clara, USA) equipped with a DB-5 capillary column (30 m × 250-μm i.d., 0.25-μm film thickness; Agilent J&W Scientific, Folsom, CA). Derivatized samples (1 μl) were injected into the DB-5 capillary column with a split ratio of 30:1 using an autoinjector. Helium was used as the carrier gas at a constant flow rate of 1 ml/min. The injection, ion source, and ion source surface temperatures were set to 300 °C, 200 °C, and 280 °C, respectively. The GC oven temperature was heated to 80 °C for 1 min, increased to 100 °C at a rate of 2 °C/min, increased to 300 °C at a rate of 15 °C/min and then maintained at 300 °C for 6 min. Electron impact ionization (70 eV) at the full scan mode (*m*/*z* 80–500) at a rate of 20 scans/s was used. Ribitol served as an internal standard to monitor batch reproducibility and to correct for minor variations that occurred during sample preparation and analysis.

### Data analysis and pattern recognition analysis

GC-MS Real Time Analysis software (Shimadzu Co., Japan) was used to process the GC-MS data. Metabolites were identified by matching the data with the NIST mass spectral library (http://www.nist.gov/srd/). After normalization against the internal standard (peak areas of ribitol) and dry weight of cells to acquire the relative abundance of metabolites, all of the peak areas of the identified metabolites were used as metabolomics profile data and then were analysed using the software from the SIMCA package 10.0 (Umetrics, Umea, Sweden).

After mean-centring and Par-scaling, the data were used for further analysis. Supervised partial least squares-discriminant analysis (PLS-DA) was initially performed to obtain an overview of the GC-MS data from different groups. To identify the metabolite changes that were associated with TCBPA exposure, a PLS-DA pairwise comparison was subsequently carried out. Variables responsible for distinguishing TCBPA-treated groups from the control group (without TCBPA) were selected by loading plots and variable importance in the projection (VIP) value threshold (VIP > 1) from the 7-fold cross-validated PLS-DA models. The accuracy and predictive ability of the models were evaluated using *R* ^2^ and *Q* ^2^ values.

The identified differential metabolites elicited by TCBPA exposure that were selected by the PLS-DA models were further analysed by another multivariate statistical analysis method, hierarchical cluster analysis (HCA), to assess the predictive accuracy of the PLS-DA models. HCA was performed using Cluster 3.0 and was visualized using TreeView 1.1.6 software.

### Reverse transcription of total RNA and quantitative real-time PCR

Genes that are responsible for encoding rate-limiting enzymes involved in carbohydrate, fatty acid and energy-related metabolism were chosen for the determination of the expression levels. For the measurement of the expression of these genes, exponential-phase (i.e., 8 h of incubation) *S*. *cerevisiae* cells were collected from the control and TCBPA-treated groups. Yeast cell pellets were quickly frozen in liquid nitrogen for RNA extraction or were stored at −80 °C until use. The total RNA in *S*. *cerevisiae* cells was extracted according to the manual for Trizol (Invitrogen, USA) and was used for the synthesis of cDNA. RNA integrity and purity were evaluated by gel electrophoresis and the OD_260_/OD_280_ ratio, respectively. cDNA was synthesized using the extracted RNA, M-MLV reverse transcriptase and random primers according to the manufacturer’s instructions (Promega, USA). cDNA from this step was stored at −20 °C for use as the template for qRT-PCR. Relative quantification of genes was performed using the Eppendorf Mastercycler® RealPlex^2^. Primers of related metabolism genes were designed by Primer Premier 5 with manual editing and are listed in the Supporting Information (Supplementary Table S2). For each amplification reaction, a total of 20.1 μl was used consisting of 2 μl of template, 10 μl of real SYBR mixtures, 0.3 μl each of the forward and reverse primer (10 μM each), and 7.5 μl of H_2_O. Amplifications were performed after an initial denaturation at 95 °C for 10 min followed by 40 cycles of PCR at 95 °C for 15 s and at 60 °C for 1 min. The melting curve of each PCR product was determined to ensure the specific amplification of the target gene. The mRNA levels of the selected genes were normalized to that of the *ACT1* gene in the same sample. The mean fold change in the abundance of transcripts was determined according to the method of Livak and Schmittgen^52^.

### Statistical analysis

An independent-sample T test was performed on specific metabolites and genes using IBM SPSS Statistics 20.0 (SPSS Inc., USA) to assess the statistical significance of the metabolic changes. Differences showing P-values less than 0.05 were considered statistically significant.

## Electronic supplementary material

Supporting Information
