# Supplementary material for: The Toxic Effects of Tetrachlorobisphenol A in Saccharomyces cerevisiae Cells via Metabolic Interference
Source: Sci Rep. 2017 Jun 1;7:2655. doi: 10.1038/s41598-017-02939-y (PMC5453934; doi:10.1038/s41598-017-02939-y)
Supplement: Supplementary file 1 — Supporting Information [file 41598_2017_2939_MOESM1_ESM.pdf]

The Toxic Effects of Tetrachlorobisphenol A in *Saccharomyces cerevisiae* Cells via Metabolic Interference

Juan Tian[1], Zhihua Ji[1], Fengbang Wang[2], Maoyong Song[2,3,*] & Hao Li[1,*]

[1] Beijing Key Laboratory of Bioprocess, College of Life Science and Technology, Beijing University of Chemical Technology, Beijing 100029, China

[2] State Key Laboratory of Environmental Chemistry and Ecotoxicology, Research Center for Eco-Environmental Sciences, Chinese Academy of Sciences, Beijing 100085, China

[3] Institute of Environment and Health, Jianghan University, Wuhan 430056, China

*Correspondence:

lihao@mail.buct.edu.cn or lihaoh@163.com (H. Li), Tel.: 0086-10-64447747, Fax: 0086-10-64416428

smsong@rcees.ac.cn (M. Song), Tel.: 0086-10-62849178, Fax: 0086-10-62849600

Supplementary Table S1 Statistical data from partial least squares-discriminant analysis (PLS-DA) at different concentrations of TCBPA.

Sample	PLS PC'S	R^2X(cum)	R^2Y(cum)	Q^2(cum)
All	3	0.764	0.749	0.615
0 vs 25 μ M	2	0.771	0.975	0.937
0 vs 50 μ M	2	0.777	0.993	0.985
0 vs 100 μ M	2	0.773	0.993	0.974

Supplementary Table S2 Oligonucleotides used in this study.

Primers	Sequences (5' to 3')
ACT1-F/R	CTCCAATGAACCCTAAATCAAACAG / CGGAAGAGTACAAGGACAAAACG
ACC1-F/R	AGATTGCGTGTTTCTTCTGCC / TGCGTTCTTGACTTCGGTGTA
CIT1-F/R	CCGTGTTAGACCCCGAAGAAGG / AGGTATTTCACCAGTCAAAAGCAACC
GCY1-F/R	CACATTGATACTGCTGCTATTTACCGT / GATTTCTTCCCGAGGAACACCTG
GPD1-F/R	CCTGGCATCACTCTACCCGACA / AGACAGGAGATAGCTCTGACGTGTGA
GPP2-F/R	CAACGCTTTGAACGCTCTACCA / GGTCTCCTGATTCCCAGATGCTC
HFA1-F/R	GACAATAACCAAGCCTGAAACG / GACAGTGCCCGCAGAAACA
HXK2-F/R	GGTTCCGTTTACAACAGATACCCAG / CAATAACAGCGGCACCAGCA
IDH1-F/R	ATGAATCCGTCCCTGGTGTAGTG / TGGCGAAGTCAAAGGCAAATC
KGD1-F/R	CACCCATTCCCATTTGCTCAG / TGTGTATGCCCACGAACCCAT
NDE1-F/R	AGTACCGATGAAACTGAGGTATCACG / TCAGAGCCGATGTACGCAAGG
NDE2-F/R	ACGCCTGGAGAAGTTCACTACATTG / TGGTTTTAGCGCCTACACTAACAACA
NDI1-F/R	ACCTCGACGAATACGCTAGTCAGA / CCAGTATCAGCACGTTTGGTTTG
OLE1-F/R	TCTACGCTATCTTCGGTTGTGCT / CCTTGTATTTTGGATTTGGCTTCA
PFK1-F/R	ATGCCACCGCTAAATCCCACT / CCAGCCATCAAGGCCAACC
PYK1-F/R	CAACGCCAGAAAGTCCGAAGA / GTGGTTTGGTGGGATTGGGTAG
YPR1-F/R	CCATTCCAGTGTTGGGTTTCG / CCCTGCCAACTTCTTCTTCATTC

Supplementary Figures

Supplementary Figure S1 Typical TICs of GC-MS from (a) the *Saccharomyces cerevisiae* control group, (b) the 25 μM TCBPA-treated group (c) the 50 μM TCBPA-treated group and (d) the 100 μM TCBPA-treated group.

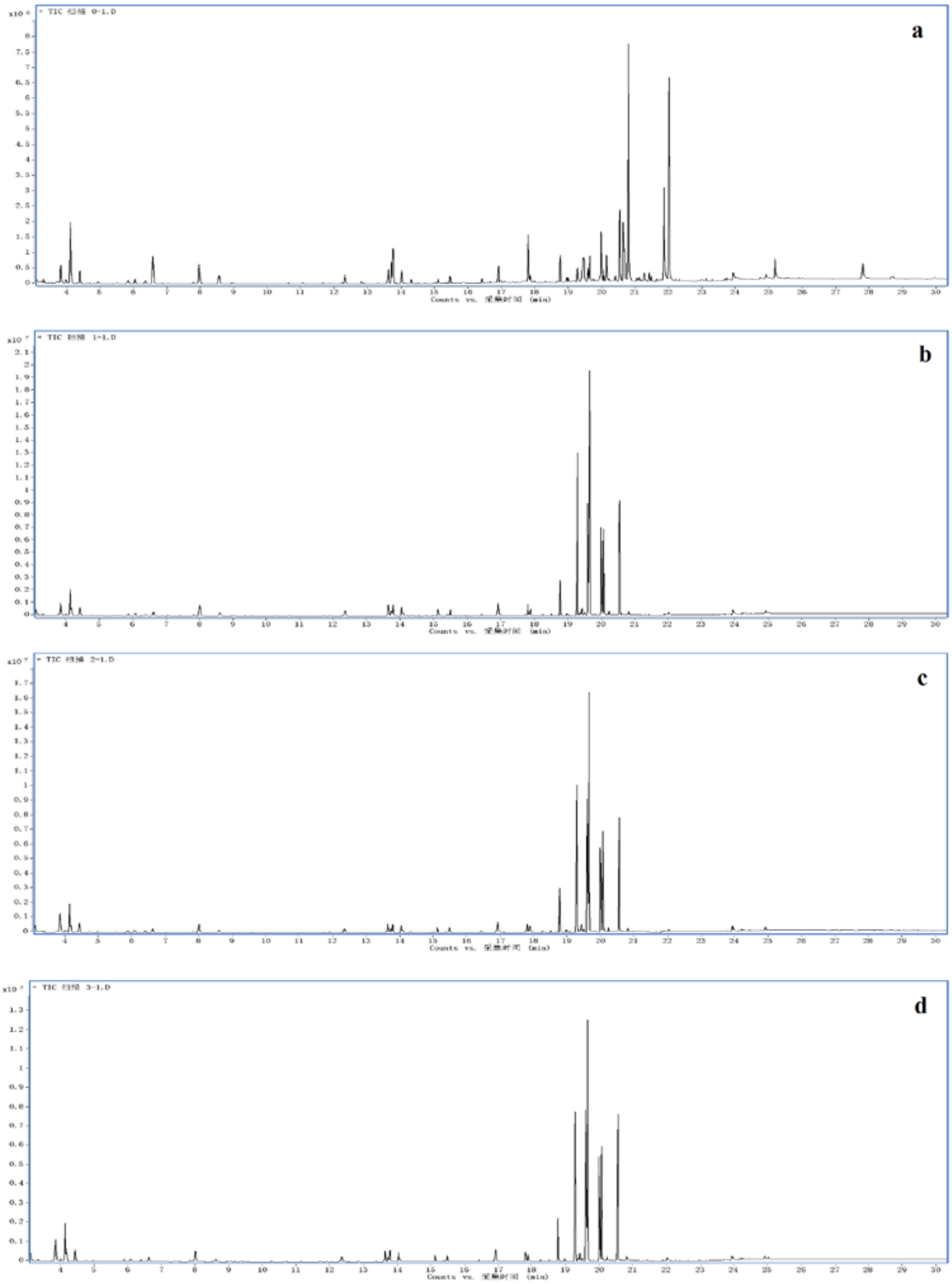

Supplementary Figure S1

Supplementary Figure S2 Scores plot for the control group and TCBPA-treated groups. (a) PCA-derived metabolites profiles. (b) PLS-DA-derived metabolites profiles. In the scores plot, the confidence interval is defined by the Hotelling's T2 ellipse (95% confidence interval), and observations outside the confidence ellipse are considered outliers. Red symbols denote samples from the control group, while green, blue and black symbols denote samples from the 25, 50 and 100 μM TCBPA treated groups, respectively.

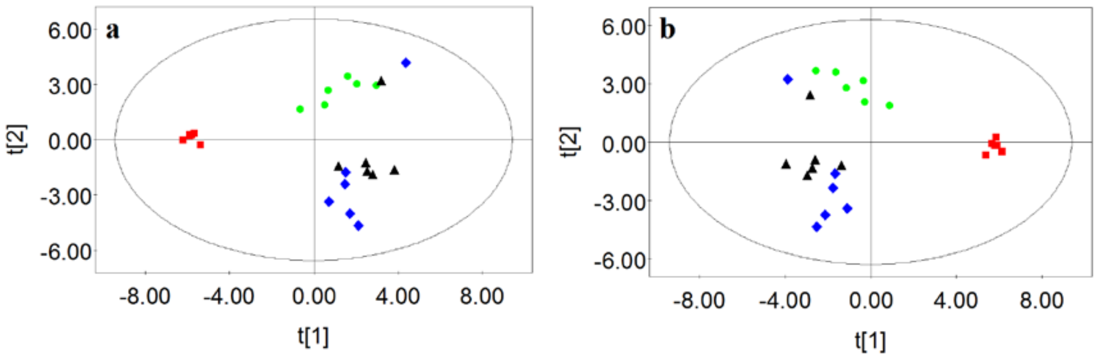

Supplementary Figure S2

Supplementary Figure S3 Variable importance of the projection plots for the intracellular metabolites along component 1. (a) Control versus 25 μM TCBPA-treated group using PLS-DA model, (b) control versus 50 μM TCBPA-treated group using PLS-DA model, (c) control versus 100 μM TCBPA-treated group using PLS-DA model.

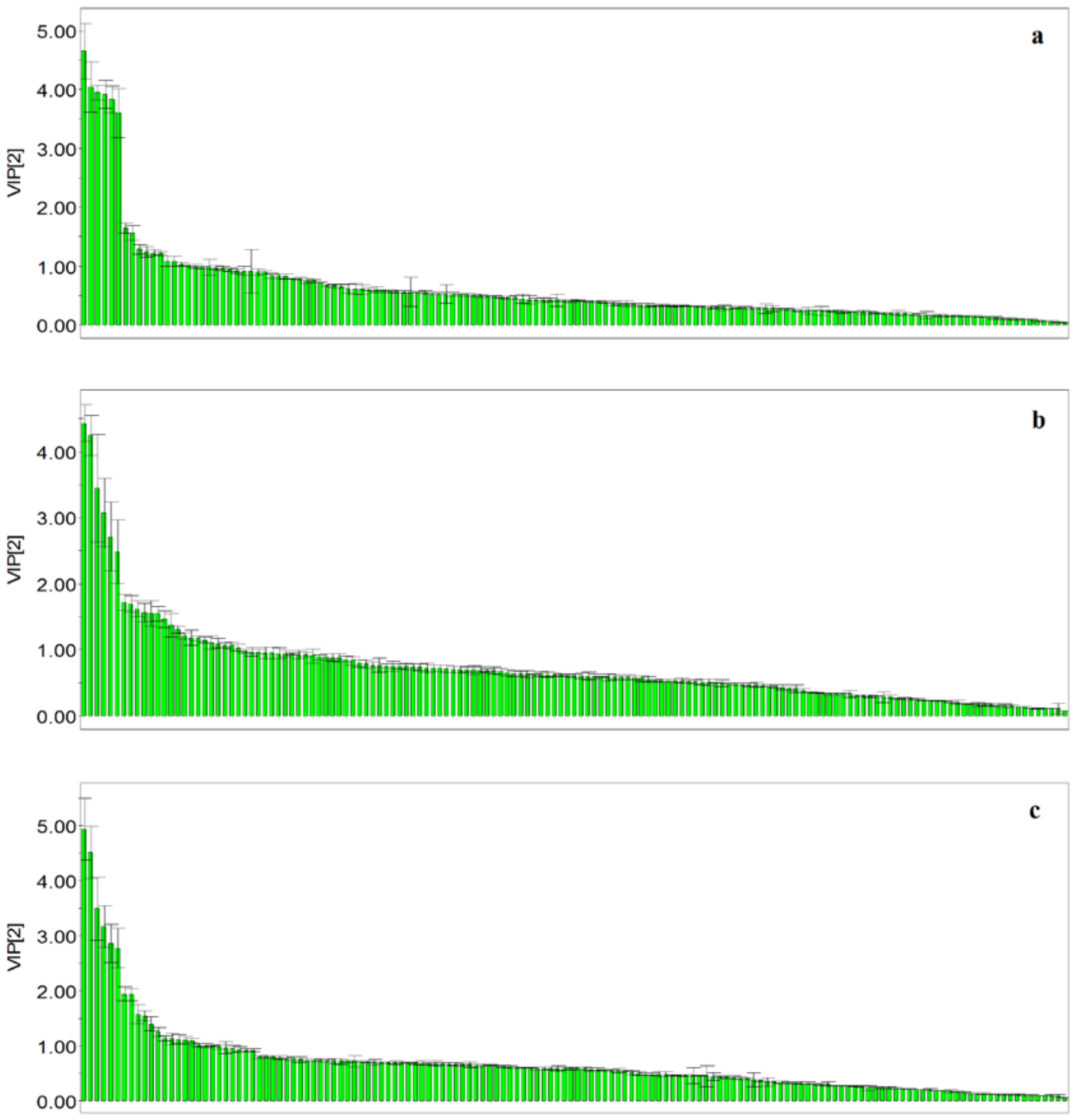

Supplementary Figure S3

Supplementary Figure S4 Hierarchical cluster analysis of the 31 identified differential metabolites.

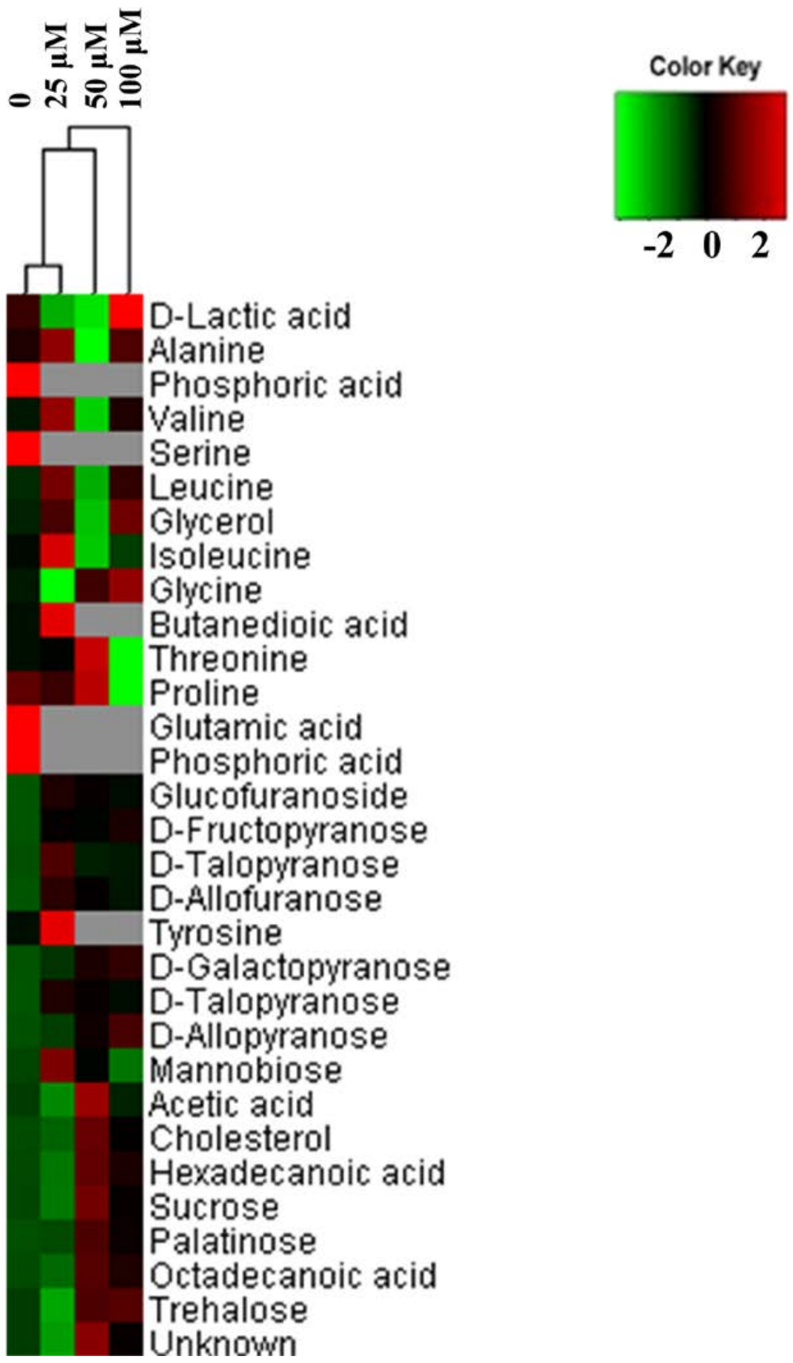

Supplementary Figure S4

Supplementary Figure S5 TCBPA induces metabolic changes in *Saccharomyces cerevisiae* as indicated by changes in metabolite and gene transcription levels. Red symbols denoted significant increases (P<0.05), and green symbols denote significant decreases (P<0.05), whereas blue symbols denote no significant changes in the metabolite levels (P>0.05). Ala, alanine; Asn, asparagine; Asp, aspartic acid; BCAA, branched chain amino acids; Gln, glutamine; Glu, glutamic acid; Gly, glycine; Ile, isoleucine; α-KG, α-ketoglutarate; Leu, leucine; Lys, lysine; Met, methionine; PEP, phosphoenolpyruvate; Phe, phenylalanine; Pro, proline; Ser, serine; Thr, threonine; Trp, tryptophan; Tyr, tyrosine; Val, valine.

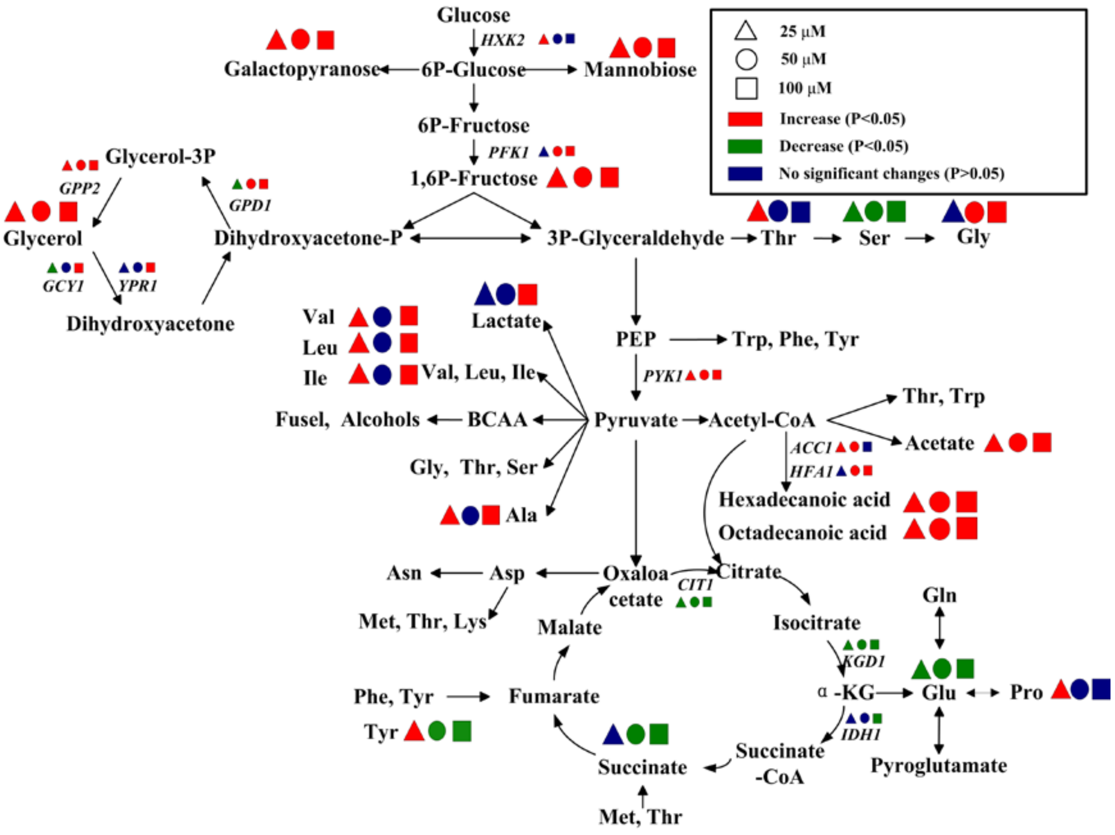

Supplementary Figure S5

Supplementary Figure S6 ROS levels in *Saccharomyces cerevisiae* control group and TCBPA-treated groups. ROS levels were detected with a fluorescent probe DCFH-DA. (a) to (i) Images scanned by Laser Scanning Confocal Microscope, (j) fluorescence intensity measured by Microplate reader.

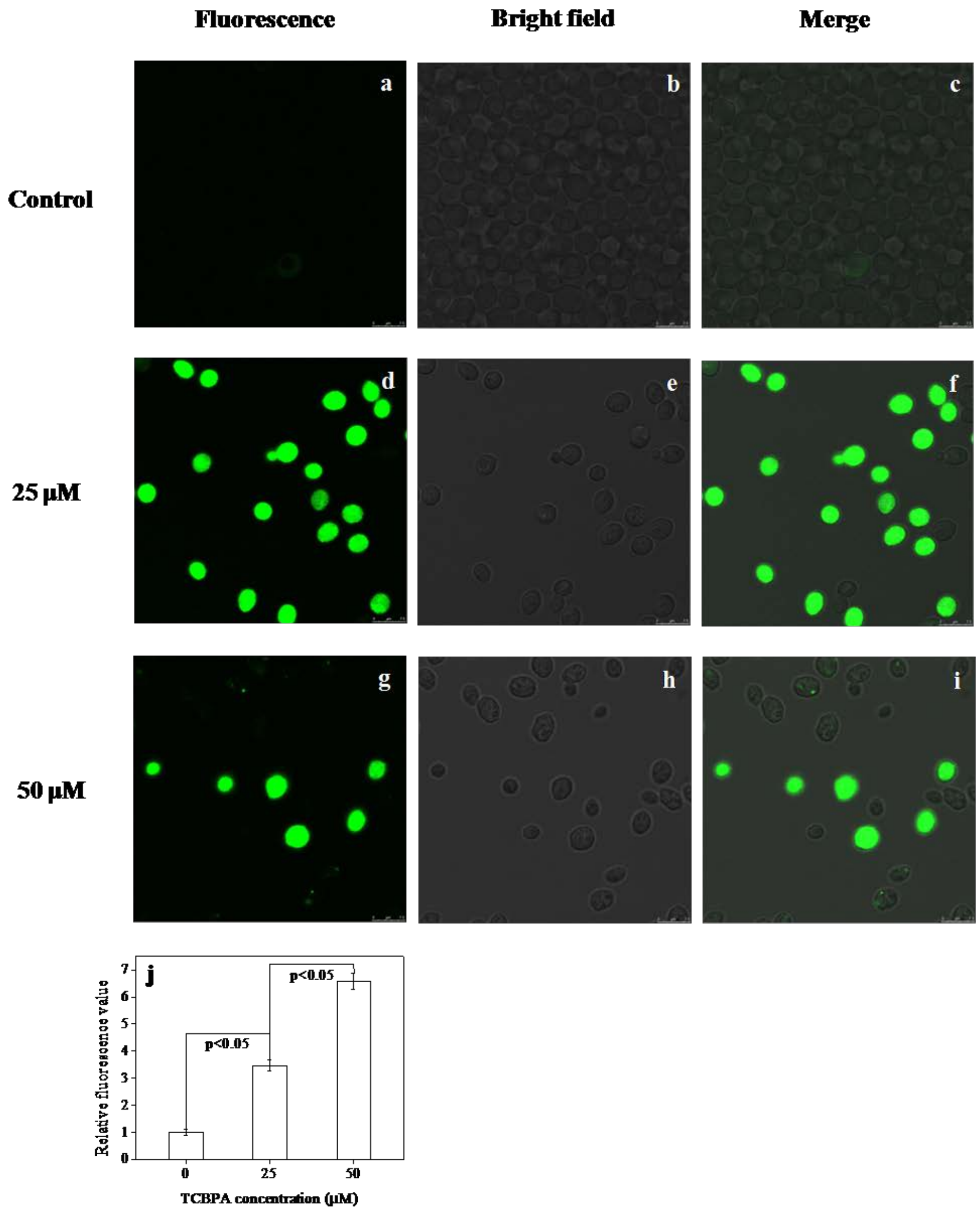

Supplementary Figure S6
